# Supplementary material for: Evaluating Beauveria bassiana Strains for Insect Pest Control and Endophytic Colonization in Wheat
Source: Insects. 2025 Mar 10;16(3):287. doi: 10.3390/insects16030287 (PMC11943200; doi:10.3390/insects16030287)
Supplement: Supplementary file 1 [file insects-16-00287-s001.zip › insects-3415923-supplementary.pdf]

Supplementary materials:

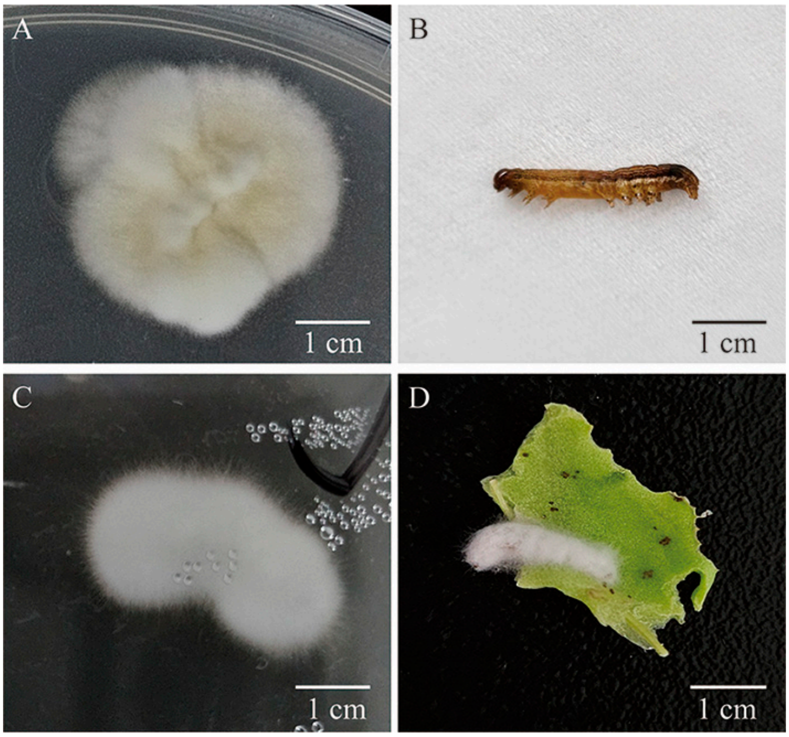

Figure S1. Stiffness of the infected larvae. Colonies were formed on PDA after incubating the dead *H. armigera* (A) and *S. frugiperda* (C) infected with *B. bassiana* CBM1 for seven days. The body color of *M. separata* larva began to darken at the first day of death (B). Colonies were growing from the surface of the *P. xylostella* larva in natural environment after seven days (D).
